# Japanese encephalitis in a 114-month-old cow: pathological investigation of the affected cow and genetic characterization of Japanese encephalitis virus isolate

**DOI:** 10.1186/1746-6148-10-63

**Published:** 2014-03-11

**Authors:** Naomi Kako, Seiji Suzuki, Norie Sugie, Tomoko Kato, Tohru Yanase, Makoto Yamakawa, Hiroaki Shirafuji

**Affiliations:** 1 Aichi Chuo Livestock Hygiene Service Center, Aichi Prefectural Government, 1-306 Jizono, Miaicho, Okazaki 444-0805, Japan; 2 Subtropical Diseases Research Division, National Institute of Animal Health (NIAH), National Agriculture and Food Research Organization (NARO), 2702 Chuzan, Kagoshima 891-0105, Japan; 3 Viral Disease and Epidemiology Research Division, NIAH, NARO, 3-1-5 Kannondai, Tsukuba 305-0856, Japan

**Keywords:** Japanese encephalitis, Cow, Arthropod-borne virus, Viral encephalomyelitis

## Abstract

**Background:**

Japanese encephalitis virus (JEV) is classified into the genus *Flavivirus* in the family *Flaviviridae*. JEV can cause febrile illness and encephalitis mainly in humans and horses, and occasionally in cattle.

**Case presentation:**

In late September 2010, a 114-month-old cow showed neurological symptoms similar to the symptoms observed in previous bovine cases of Japanese encephalitis (JE); therefore, we conducted virological and pathological tests on the cow. As a result, JEV was isolated from the cerebrum of the affected cow. We determined the complete genome sequence of the JEV isolate, which we named JEV/Bo/Aichi/1/2010, including the envelope (E) gene region and 3’ untranslated region (3’UTR). Our phylogenetic analyses of the E region and complete genome showed that the isolate belongs to JEV genotype 1 (G1). The isolate, JEV/Bo/Aichi/1/2010, was most closely related to several JEV G1 isolates in Toyama Prefecture, Japan in 2007–2009 by the phylogenetic analysis of the E region. In addition, the nucleotide alignment revealed that the deletion in the 3’UTR was the same between JEV/Bo/Aichi/1/2010 and several other JEV G1 isolates identified in Toyama Prefecture in 2008–2009. A hemagglutination inhibition (HI) test was conducted for the detection of anti-JEV antibodies in the affected cow, and the test detected 2-mercaptoethanol (2-ME)-sensitive HI antibodies against JEV in the serum of the affected cow. The histopathological investigation revealed nonsuppurative encephalomyelitis in the affected cow, and the immunohistochemical assay detected JEV antigen in the cerebrum.

**Conclusion:**

We diagnosed the case as JE of a cow based on the findings of nonsuppurative encephalomyelitis observed in the central nervous system, JEV antigen detected in the cerebrum, JEV isolated from the cerebrum, and 2-ME-sensitive HI antibodies against JEV detected in the serum. This is the first reported case of JE in a cow over 24 months old.

## Background

Japanese encephalitis virus (JEV) is a member of the genus *Flavivirus* in the family *Flaviviridae*, and it is distributed throughout eastern, southern and southeastern Asia, Papua New Guinea, and the Torres Strait of northern Australia [1]. JEV is transmitted by mosquitoes and amplified in pigs and wading birds in nature. It can also infect humans, horses, cattle and many other vertebrates [2,3]. Although the titers of viremia in JEV-infected humans, horses and cattle seem not to be high enough for infecting mosquitoes [2,4,5], JEV can cause febrile illness and encephalitis, mainly in humans and horses [1-3,6], and occasionally in cattle [7-9]. A case of nonsuppurative encephalomyelitis occurred recently in a 141-day-old calf in Miyazaki Prefecture, Japan, and JEV genotype 1 (G1) was isolated from the cerebrum of the affected calf, suggesting that JEV G1 can cause neurological disorders in cattle [10]. In late summer of the following year, a 114-month-old cow in Aichi Prefecture, Japan showed clinical symptoms similar to those of the calf; we therefore conducted virological and pathological tests on the cow.

## Case presentation

### Sample collection

In late September 2010, a 114-month-old Japanese Black cow on a beef farm in Aichi Prefecture, Japan showed decreased appetite and fever. Four days after the onset of these symptoms, the cow showed astasia. Despite palliative treatments, no improvement was seen, and the cow then became recumbent and was euthanized. A serum sample was collected after the onset of the initial symptoms, and organs were collected at necropsy for virological and pathological investigations including the brain, spinal cord, heart, lung, liver, kidney, spleen, rumen, reticulum, omasum, abomasum, intestines, right axillary lymph node, and right popliteal lymph node. Serum samples were collected from the other 14 cows on the farm for anti-JEV hemagglutination inhibition (HI) antibody.

### Virological, serological and pathological investigations

Homogenate was prepared from the cerebrum of the affected cow, and RNA was extracted from the homogenate and the cow’s serum sample with a QIAamp Viral RNA Mini Kit (Qiagen, Valencia, CA, USA). The RNA was tested for the presence of JEV by reverse transcription-polymerase chain reaction (RT-PCR) [11]. For virus isolation, the homogenate was inoculated onto Vero cells [6], and the isolated virus was subjected to RT-PCR and direct sequencing using both primer sets targeting the envelope (E) gene region and the 3’ untranslated region (3’UTR), respectively [12]. The complete genome sequence was determined by a direct sequencing using the products of RT-PCR, 5’RACE, and 3’RACE as sequencing templates [10]. All of the primers used in the present study are shown in Table 1.

**Table 1:** Oligonucleotide primers used for the cDNA amplification and sequencing of JEV/Bo/Aichi/1/2010

**Primer**	**Sequence (5'-3')**	**Position**	**Purpose**	**References**
JF1	TTACTCAGCGCAAGTAGGAGCGTCTCAAG	1440-1468	RT-PCR (E region)	Yeh *et al*. (2010)
JF2	TTACTCAGCGCAAGTTGGGGCGTC	1440-1463		
JR1	ATGCCGTGCTTGAGGGGGACG	1675-1655		
JR2	CAYGCTGTGCTCGAAGGGGACG	1675-1654		
JE955f	TGYTGGTCGCTCCGGCTTA	956-974	RT-PCR (E region)	Nerome *et al*. (2007)
JE2536r	AAGATGCCACTTCCACAYCTC	2537-2517		
JE10141f	TGGATTGAAGAAAATGAATGGATG	10141-10164	RT-PCR (3'UTR)	Nerome *et al*. (2007)
JE10965r	AGATCCTGTGTTCTTCCTCTC	10965-10945		
JEV9-2193F	ATCTGTGTGAACTTCTTGGC	9-28	RT-PCR and sequencing	Katayama *et al. *(2013)
JEV9-2193R	TTTACCCAGCGTGCTTCCAGC	2193-2173		
JEV1850-3845F	TGGACAAACTGGCTCTGAAGGG	1850-1871		
JEV1850-3845R	TTCTCTTGGTTCGTCCATCTCG	3845-3824		
JEV3655-5606F	CTACTTGTGCTGATGCTTGG	3655-3674		
JEV3655-5606R	ATTGGGGCATTTGAGTC	5606-5590		
JEV5409-7958F	CCATAGACTAATGTCACCAAAC	5409-5430		
JEV5409-7958R	AGAGTTGCTGCGTAGTAG	7958-7941		
JEV7543-9421F	GACAATGGAGCCAGTGC	7543-7559		
JEV7543-9421R	CCTTGACCACTTTGTGCCTG	9421-9402		
JEV9234-10965F	CATTCTCCGTGACATAGCAGG	9234-9254		
JEV9234-10965R	AGATCCTGTGTTCTTCCTCACC	10965-10944		
JEV1108R	GGACATCTAGTGTTGGTTTG	1108-1089	5'RACE and sequencing	Katayama *et al.* (2013)
JEV1030R	CTCCACTGGCTCCTTCTATG	1030-1011		
JEV157R	ATCCGCGTTTCAGCATATTGATGG	157-134		
JEV9568F	GTCATCGGACCACAACACTTG	9568-9588	3'RACE and sequencing	Katayama *et al.* (2013)
JEV10757F	CCGTGGAAACAACATTATGC	10757-10776		
JEV3305R	TCAAGGACAATGCCGTTCTC	3305-3286	Sequencing	Katayama *et al.* (2013)
JEV5124R	CTCTTGACGGTCGCCTTGC	5124-5106		
JEV7367R	TCCACGACGGCATTCTTCATTAT	7367-7345		
JEV7819F	AACATAGTGGGAGGACATC	7819-7837		
JEV8993R	GCATTGACCATCTCCCAGAAC	8993-8973		
JEV10306R	CCCTCACTTGGTTTATTGCCG	10306-10286		
JEV970R	CTGGAGCGACCAATAGCAAG	970-951	Sequencing	This study
JEV2240F	GTGACACAGCCTGGGACTTC	2240-2259		
JEV3906F	AGTCCACGGAATCCTGAATG	3906-3925		
JEV6041F	CCACCAGTGAAGATGACAGCAAC	6041-6063		
JEV7539R	CAAGGTGAGTGTGGCTGCCGTC	7539-7518		
JEV8090F	AGCCTAGTGACACCCTGTTC	8090-8109		
JEV9249R	TATGTCACGGAGGATGTATC	9249-9230		

To analyze the phylogenetic relationships among virus isolates, we aligned sequences using the Clustal W program [13], and we constructed phylogenetic trees with MEGA5 using the neighbor-joining method. The reliability of the branching orders was evaluated by the bootstrap test (*n* = 1,000) [14]. The nucleotide sequence data reported in this study were deposited in the DNA Data Bank of Japan (DDBJ) with the accession numbers AB797319 (E region), AB797320 (3’UTR) and AB853904 (complete genome). We also tested the affected cow for bovine spongiform encephalopathy (BSE) by enzyme-linked immunosorbent assay (ELISA) (NippiBL, Nippi, Tokyo).

A hemagglutination inhibition (HI) test was conducted to determine the anti-JEV HI antibody titers of the affected cow and all other cows at the farm [6]. Prior to the test, all of the serum samples were treated with acetone and then mixed with goose red blood cells. The HI antibody titers were determined with the use of JEV antigen prepared from the Nakayama strain (Kyoto Biken, Kyoto, Japan). JEV-positive swine serum was used as a positive control. We also treated the serum sample of the affected cow with 2-mercaptoethanol (2-ME) and then subjected it to the HI test, to test for anti-JEV immunoglobulin M antibodies.

We examined the affected cow for gross and histological lesions. The collected organs were fixed in 10% neutral buffered formalin. Fixed organs were then embedded in paraffin wax, sectioned at 3 μm, and stained with hematoxylin and eosin. An immunohistochemical (IHC) assay was performed using a Histofine Simple Stain MAX-PO (MULTI) Kit (Nichirei, Tokyo). Anti-JEV (AS-6 strain [15]) polyclonal rabbit serum was used as the primary antibody for the detection of JEV antigen.

### Isolation and genetic characterization of JEV

**Figure 1:**
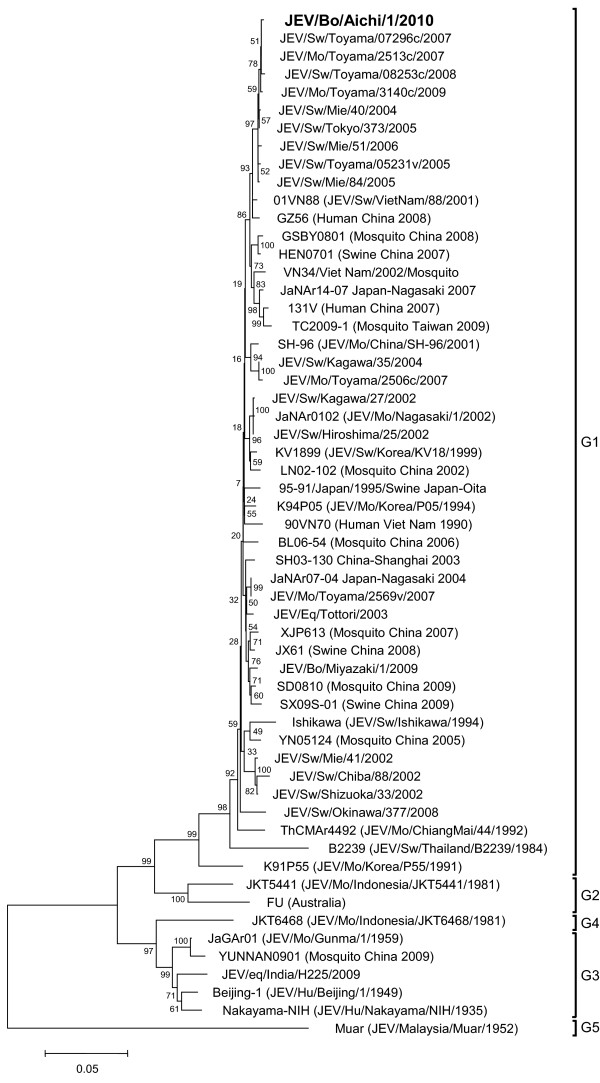
**Phylogenetic profile showing the relationships among JEV isolates based on a comparison of the E gene.** The percentage bootstrap values calculated from 1,000 replications are indicated around the internal nodes. The scale represents 0.05% sequence divergence. G1–G5: genotypes 1–5.

The cerebrum of the affected cow tested positive for JEV, but the serum tested negative. We then subjected the cerebrum to virus isolation, and JEV was isolated after the second passage. From the JEV isolate, which we named JEV/Bo/Aichi/1/2010, cDNAs containing the E region and the 3’UTR were successfully amplified and sequenced. Our phylogenetic analysis of the E region showed that the isolate clustered with other isolates belonging to JEV G1 (Figure 1), and it was most closely related to several isolates from Toyama Prefecture, Japan in 2007–2009 [16], including JEV/Sw/Toyama/08253c/2008, JEV/Sw/Toyama/08253c/2008, JEV/Sw/Toyama/07296c/2007 and JEV/Mo/Toyama/3140c/2009.

**Figure 2:**
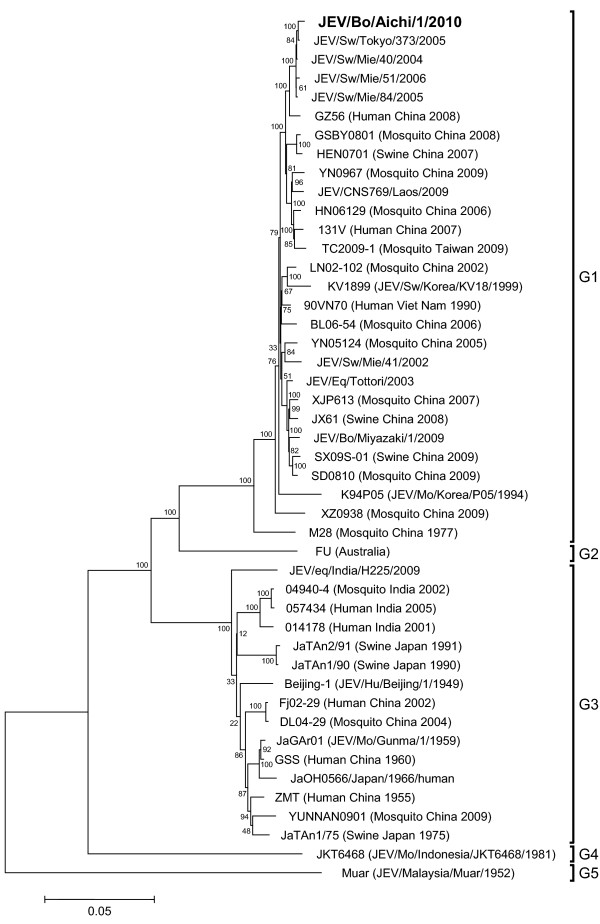
**Phylogenetic profile showing the relationships among JEV isolates based on a comparison of the complete genome.** The percentage bootstrap values calculated from 1,000 replications are indicated around the internal nodes. The scale represents 0.05% sequence divergence. G1–G5: genotypes 1–5.

**Figure 3:**
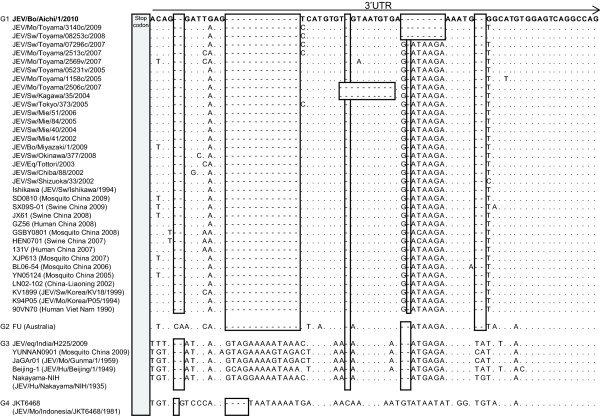
**Nucleotide sequence alignment of the 5’ distal region of the 3′ UTRs.** The nucleotide sequences of the 3′ UTR were compared between the JEV isolate characterized in this study (JEV/Bo/Aichi/1/2010) and other isolates of genotypes 1–5 collected elsewhere, available from GenBank. The sequence of JEV/Bo/Aichi/1/2010 is shown in **bold**, and only differences from the sequence are indicated for other JEV isolates. Sequence gaps were introduced to optimize the alignment, and deletions are indicated by hyphens and open boxes.

The newly identified JEV isolate, JEV/Bo/Aichi/1/2010, was found to contain 10,958 nucleotides, and our phylogenetic analysis of the complete genome also showed that the isolate was closely related with other isolates of JEV G1 (Figure 2). The nucleotide sequence of the 3’UTR was then compared between JEV/Bo/Aichi/1/2010 and other JEV isolates belonging to genotypes 1 - 5 available in GenBank. The nucleotide alignment revealed that JEV/Bo/Aichi/1/2010 has the same deletion in the 3’UTR as several other isolates of JEV G1 in Toyama Prefecture, such as JEV/Mo/Toyama/3140c/2009 and JEV/Mo/Toyama/08253c/2008, although their sequences were not completely identical (Figure 3). The affected cow was negative for BSE by the ELISA.

### JEV seroprevalence

We conducted HI tests for the affected cow and the other healthy cows on the farm to determine the serological prevalence of JEV infection. All 15 of the cows tested positive for anti-JEV HI antibodies, and the titers were 1:160 in the affected cow and ranged from 1:160 to 1:2560 in the other cows (Table 2). In addition, the affected cow had 2-ME sensitive HI antibody, and the titer was 1:20.

**Table 2:** Anti-JEV HI titers of serum samples collected from all the cows in the farm, located in Aichi Prefecture, Japan

**Cow no.**	**Age (month)**	**HI titer**
1	29	1:160
2	33	1:160
3	35	1:640
4	58	1:320
5	68	1:640
6	80	1:640
7	81	1:640
8	90	1:640
9	100	1:1280
10*	114	1:160
11	115	1:640
12	143	1:640
13	147	1:640
14	148	1:320
15	188	1:2560

*The affected cow.

### Pathological findings

**Figure 4:**
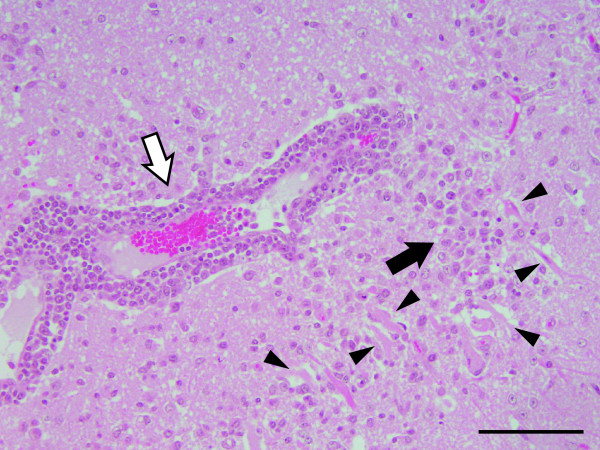
**Neuronal necrosis, a glial nodule and perivascular infiltration of lymphocytes in the cerebral cortex of the affected cow.** Arrowheads point out the neuronal necrosis. Black arrows: the glial nodules; white arrows: the perivascular infiltration of lymphocytes. Hematoxylin and eosin (H&E) stain. Bar = 100 μm.

**Figure 5:**
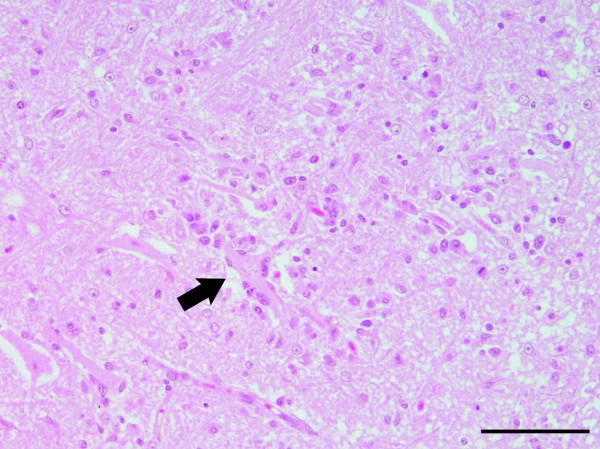
**Neuronophagia in the cerebral cortex of the affected cow.** The arrow indicates the neuronophagia. H&E stain. Bar = 100 μm.

The gross examination of the affected cow revealed hemorrhage and congestion of the brain, pulmonary emphysema, rough surface of the kidney, swelling of the right axillary and right popliteal lymph nodes, and a wound and muscle necrosis of the right upper limb. The histological examination revealed typical nonsuppurative encephalomyelitis. Perivascular infiltration of lymphocytes was widely distributed throughout the cerebrum (Figure 4), and neuronal necrosis, neuronophagia and glial nodules were observed in the cerebral cortex (Figures 4 and 5).

**Figure 6:**
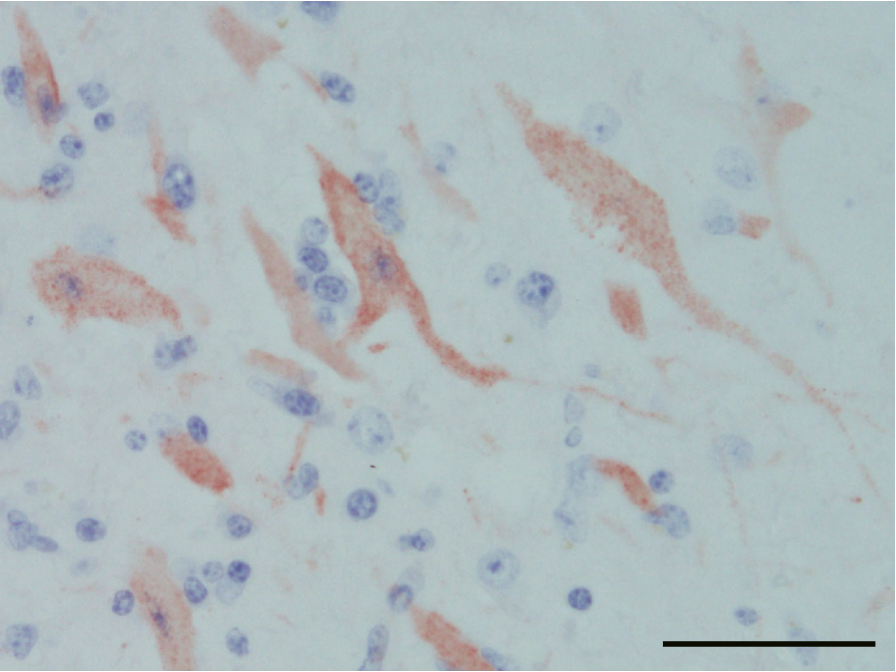
**The IHC assay results.** Detection of JEV antigen in the cerebrum of the affected cow. JEV-positive granules (brown color) were observed in the cytoplasm of neurons and in the nerve fibers. Bar = 50 μm.

These histological lesions were also observed in the medulla oblongata and spinal cord, and neuronal necrosis was observed in the diencephalon. The lesions were most severe in the hippocampus and were also severe in the frontal lobe among the affected sites in the central nervous system (CNS). Histological findings in other organs included *Sarcocystis* spp. infection in the myocardium, mild abomasal ulceration, mild nonsuppurative interstitial nephritis, severe diffuse hemosiderosis in the spleen, and infiltration of neutrophils in the right axillary and right popliteal lymph nodes. The IHC assay detected JEV antigens in the cerebrum. JEV-positive granules were observed in the cytoplasm of neurons and in the nerve fibers (Figure 6).

## Discussion

We diagnosed the case as JE of a cow based on the following findings: nonsuppurative encephalomyelitis observed in the CNS, JEV antigen detected in the cerebrum, JEV isolated from the cerebrum and 2-ME sensitive HI antibodies against JEV in the serum. The histopathological findings observed in the CNS of the affected cow were similar to those observed in the 141-day-old calf in the previous JE case [10], including perivascular infiltration of lymphocytes, neuronal necrosis, neuronophagia and glial nodules.

However, two other lesions observed in the present case were not observed in the 141-day-old calf but were seen in other bovine JE cases: pulmonary emphysema was observed in a 24-month-old milking cow in 1950, and congestion of the brain was seen in an 18-month-old Japanese Black cow in 1996 [7,9]. It is possible that the pulmonary emphysema—as well as the mild abomasal ulceration and mild nonsuppurative interstitial nephritis—observed in the present study influenced the immune status of the affected cow, and contributed to the invasion of JEV into the CNS. The congestion of the brain may have been caused by the onset of encephalomyelitis, since similar gross lesions were observed in some human JE cases [17,18].

The affected cow’s wound and muscle necrosis of the right upper limb seemed to have resulted from the onset of astasia and recumbency, and the infiltration of neutrophils in the right axillary and right popliteal lymph nodes may have been immune responses to wound infections. However, it is unclear whether the other pathological findings in the affected cow had relevance to the onset of JE, such as the severe diffuse hemosiderosis in the spleen and the infection of *Sarcocystis* spp. in the myocardium, although some species of *Sarcocystis* protozoan parasites are highly pathogenic to ruminants [19].

Our genetic analyses revealed that JEV/Bo/Aichi/1/2010 was most closely related to several other JEV G1 isolates in Toyama Prefecture, which is located approx. 100 miles away from Aichi Prefecture. The JEV isolate JEV/Bo/Aichi/1/2010 was most closely related to several JEV G1 isolates in Toyama Prefecture identified in 2007–2009 by phylogenetic analysis of the E region [16]. In addition, the deletion patterns in the 3’UTR were the same between JEV/Bo/Aichi/1/2010 and some of the JEV G1 isolates found in Toyama Prefecture in 2008–2009.

There were some differences in the phylogenetic characteristics between JEV/Bo/Aichi/1/2010 and another JEV isolate from an affected calf in Miyazaki Prefecture [10], which is located approx. 400 miles away from Aichi Prefecture. Our present findings suggest that JEV/Bo/Aichi/1/2010 may be a part of the JEVs overwintering in the Chubu region of Japan [16], a region that consists of Aichi, Toyama and several other prefectures.

In the bovine JE cases reported to date, the ages of onset were 141 days, 6 months, 18 months and 24 months [7-10]; the 114-month-old affected cow in the present study was the oldest cow affected by JEV infection. The advanced age may have influenced the immune status of the affected cow. In addition, heat stress may have triggered the onset of JE in the extraordinarily hot summer of 2011. However, none of the other cows (including a 188-month-old cow on the same farm) showed any neurological signs, even though the HI test provided evidence of JEV infection in the herd. Therefore, advanced age seems not to be an important factor in the onset after JEV infection in cattle.

## Conclusions

A 114-month-old cow showed several clinical signs including decreased appetite, fever, astasia and recumbency in the summer of 2010 in Aichi Prefecture, Japan. The histopathological investigation revealed nonsuppurative encephalomyelitis in the affected cow, and JEV antigen was observed in the lesions. We isolated JEV from the cerebrum, and we thus diagnosed the case as JE of a cow. Our genetic analyses revealed that the isolate was closely related to other JEV G1 isolates from pigs and mosquitoes in Toyama Prefecture, which is approx. 100 miles away from Aichi Prefecture. Although our data clearly indicated that the causative agent was JEV in the cow’s case, the causes of the onset are still unclear, since JEV infection usually results in an asymptomatic infection in cattle. Further study is needed to clarify the actual causes of the onset after JEV infection in cattle.

### Additional information

Alignments and phylogenetic trees created in the present study are available from the Dryad Digital Repository (https://datadryad.org/resource/doi:10.5061/dryad.s0c34).

## Competing interests

The authors declare that they have no competing interests.

## Authors’ contributions

NK and SS performed the pathological and virological investigations, respectively. NK, SS and NS made the diagnosis of the cow’s case. TK, TY, MY and HS performed the genetic and phylogenetic analyses of the JEV isolate. NK and HS wrote the manuscript, and all authors read and approved the final manuscript.
